# A Fusion Method for Combining Low-Cost IMU/Magnetometer Outputs for Use in Applications on Mobile Devices

**DOI:** 10.3390/s18082616

**Published:** 2018-08-09

**Authors:** Photis Patonis, Petros Patias, Ilias N. Tziavos, Dimitrios Rossikopoulos, Konstantinos G. Margaritis

**Affiliations:** 1 Laboratory of Photogrammetry and Remote Sensing, School of Rural & Surveying Engineering, Aristotle University of Thessaloniki, Univ. Box 439, GR-54 124 Thessaloniki, Greece; patias@auth.gr; 2 Department of Geodesy and Surveying, School of Rural & Surveying Engineering, Aristotle University of Thessaloniki, GR-54 124 Thessaloniki, Greece; tziavos@auth.gr (I.N.T.); rossi@auth.gr (D.R.); 3 Department of Applied Informatics, School of Information Sciences, University of Macedonia, GR-54 636 Thessaloniki, Greece; kmarg@uom.gr

**Keywords:** low cost, inertial sensors, magnetometers, mobile device, 3D orientation, augmented reality

## Abstract

This paper presents a fusion method for combining outputs acquired by low-cost inertial measurement units and electronic magnetic compasses. Specifically, measurements of inertial accelerometer and gyroscope sensors are combined with no-inertial magnetometer sensor measurements to provide the optimal three-dimensional (3D) orientation of the sensors’ axis systems in real time. The method combines Euler–Cardan angles and rotation matrix for attitude and heading representation estimation and deals with the “gimbal lock” problem. The mathematical formulation of the method is based on Kalman filter and takes into account the computational cost required for operation on mobile devices as well as the characteristics of the low-cost microelectromechanical sensors. The method was implemented, debugged, and evaluated in a desktop software utility by using a low-cost sensor system, and it was tested in an augmented reality application on an Android mobile device, while its efficiency was evaluated experimentally.

## 1. Introduction

Nowadays, low-cost inertial measurement units (IMUs) and magnetometer sensors of microelectromechanical systems (MEMS) technology are produced massively and are available at low cost. In addition, their size is constantly shrinking and many manufacturers of electronic devices incorporate them into their products to exploit their capabilities. Examples include mobile devices and video game consoles, where developers take advantage of these sensors to create innovative applications. It should be noted that mobile devices are able to instantly calculate pose estimation using their integrated sensors. In this way, these devices have the potential to be used in geomatics and augmented reality (AR) applications [1,2,3].

Mobile devices use low-cost MEMS sensors in a strap-down layout, where all the sensors are placed on a flat area, ensuring that the corresponding axes of the different sensor systems are parallel to each other [4,5,6]. The raw data acquired by the sensors cannot be used directly in mathematical formulas for the estimation of attitude and heading representation parameters [7]. Measurement noise, along with other errors, gives results with large deviations, which, in the visual representation, leads to graphic oscillations around the correct values. MEMS sensors are mainly influenced by thermal and electronic noise, usually modeled as additive white Gaussian noise [8]. In addition, there are possible errors due to the manufacture and assembly of the sensor system [9]. The quality of the sensors can be assured by analyzing large measurement samples [4,10], and their performance can be greatly improved by applying corrections that emerge from the calibration procedure [5,9,11,12,13,14]. In this way, the accuracy that can be achieved by these systems can be enhanced by one order of magnitude.

However, even the calibrated outputs by the accelerometer sensors are not directly usable in all cases. When the force exerted on the inertial measurement unit is not the force due to gravity, but rather the result of many forces including gravity, then the results are not accurate. Even if the accelerometer sensors are in static condition, they are still very sensitive to vibration and generally to mechanical noise. On the other hand, magnetometers are not inertial sensors and their outputs depend significantly on the additional magnetic fields that are present in the operating environment. For this reason, these sensors cannot always exhibit the same performance with regard to the accuracy that can be achieved [15]. Furthermore, the outputs of low-cost magnetometers show large variance in the estimated results. Finally, gyroscopes are inertial sensors that measure the rate of angle change around the three axes of the sensor system. Gyroscopes are not free from noise; however, they are less sensitive to linear mechanical movements, to which accelerometer sensors are prone, and are not influenced by external factors, in contrast to magnetometers.

The mathematical model chosen to represent the 3D orientation of the sensor system axes may have weaknesses and limitations, which are mainly related to singularities and nonlinearity of equations in kinematic mode. These drawbacks can lead to discontinuity or unstable results in the representation of the 3D orientation. This can be crucial for the seamless execution of the application where these mathematical models are used [16].

Although modern mobile devices have abundant computing power, the demand for large computing frequency and battery consumption force developers to limit in any way the computational power required to run their applications. Therefore, the methods employed to fuse outputs by inertial and magnetometer sensors that are intended to be used in real-time applications on mobile devices must be designed in such a way that they will have the lowest possible computational cost. Additionally, it must be considered that each sensor type has different characteristics and operates at a different frequency rate.

Different approaches are available in the literature regarding the design of methods for fusing inertial and magnetic sensors [17,18,19,20,21,22,23]. The algorithms that are frequently employed include Kalman filter [17], complementary filter [21], and particle filter [22], providing optimal results. The most commonly used approaches utilize nonlinear versions of the Kalman filter to combine the outputs of sensors and the quaternions for the 3D orientation representation. The present study benefitted from the investigations presented in [23,24]. The research in [23] uses a mathematical model with Euler angles as unknown parameters, and the fusion of sensors is achieved by utilizing the Kalman filter approach. This study provides satisfactory results, but the “gimbal lock” problem is not addressed. On the other hand, the research described in [24] uses the rotation matrix to maintain the 3D orientation, and the fusion of sensor outputs is carried out by individual sensor measurements and simple techniques. 

The scope of this paper is to create a method that combines the outputs from built-in sensors of mobile devices providing the optimal results of the 3D orientation in real time. As far as the fusion method is concerned, an attempt is made to use a linear model with minimum unknown parameters, in this way reducing the computational complexity. Specifically, only some elements of the rotation matrix are used in Kalman filter in order to update the navigation parameters. In this approach, the representation method is a combination of Euler–Cardan angles and rotation matrix, bypassing the “gimbal lock” problem and considering the limited computing power of mobile devices. The method was implemented, debugged, and evaluated in a desktop software utility by using a low-cost IMU, which also included magnetometer sensors. The performance of the method was finally tested in an AR application on a mobile device powered by Android. Finally, it should be noted that the present study focuses mainly on the quality of the fusion method, its efficient implementation in AR applications, and the repeatability of the visualization results, given the accuracy limitations caused by the use of low-cost sensors. 

Regarding the structure of the paper, after this introductory section, the fusion method of combining outputs from inertial sensors and magnetometers is presented. Next, the performance of the method on the desktop utility and on the AR application developed is evaluated and discussed. Finally, the conclusions derived from this study are concisely drawn.

## 2. Method

### 2.1. Estimation of 3D Orientation Using Accelerometer and Magnetometer Sensor Output

A system of 3-accelerometer inertial sensors in a 3-orthogonal layout can estimate, in a static condition, the vector components of gravity acceleration by measuring the force that the gravitational field pulls into the reference mass of the accelerometer’s mechanism [25]. The gravity acceleration vector for the same place and for a short period of time can be considered to have a fixed value regardless of the system orientation. In this way, it is possible to calculate the 2 tilt angles with respect to the horizontal plane or the pitch and roll angles used in navigation [26]. On the other hand, determining the system’s heading in non-kinematic conditions or when a Global Navigation Satellite System receiver is not available can be achieved with the help of a 3-magnetometer sensor system in a 3-orthogonal layout that measures the intensity of the magnetic field. The projection of these axes’ intensity components to the horizontal level with the help of the pitch and roll angles leads to the system’s heading estimation [27]. The 3D orientation and representation methods that are frequently used in geomatics applications are the Euler–Cardan angles, the rotation matrix, and the quaternions [28]. Euler–Cardan angles describe the orientation of a rigid body with respect to a fixed coordinate system and constitute a 3-parameter representation method. The particular method has a major disadvantage, a mathematical singularity called the “gimbal lock” problem. Due to this singularity, when the *X*-axis is approximately vertical, then the results for the representation of the 3D system’s orientation are unstable. In case the *X*-axis of the system becomes vertical, then there is no solution at all [29]. Therefore, as it is, this method can only work efficiently provided that the *X*-axis will never get values around 90° relative to the horizontal level. On the other hand, the rotation matrix, also called the direction cosine matrix (***DCM***), and the quaternions are representation methods that use 9 and 4 parameters, respectively, therefore do not suffer from the “gimbal lock” problem [16] and are functional in all cases.

In geomatics and AR applications, the attitude and heading representations of the sensor system axes cannot have values that present abnormal changes or unstable solutions or no solution at all; therefore, it is obligatory to fuse the outputs of all necessary sensors by using refined techniques. The method developed and described in this paper combines the Euler–Cardan angles and the rotation matrix. Specifically, the rotation matrix constitutes a means of maintaining the information of the system’s orientation, while the Euler–Cardan angles are used in a specialized way to correct the rotation matrix by using the output of the sensors. The main purpose of the method is to avoid the “gimbal lock” situation, where, due to the physical loss of one degree of freedom in the 3D space, the solution for the orientation of the system is unstable or there is no solution at all.

**Figure 1:**
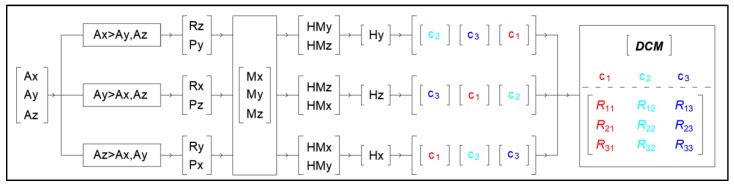
Analytical flowchart for calculating direction cosine matrix (***DCM***) by using accelerometer and magnetometer output.

The method generalizes the conventional sense of the navigation angles roll (Rx, Ry, Rz), pitch (Px, Py, Pz), and heading (Hx, Hy, Hz) and uses them selectively, depending on which axis of the inertial measurement unit is upward or has the largest absolute acceleration value, as presented in the flowchart of Figure 1.

If, for example, the *Y*-axis has the largest acceleration value, then pitch Pz and roll Rx are calculated by using the accelerometer outputs Ax, Ay, and Az. Next, the projections HMx and HMz to the horizontal level of the *X-* and *Z*-axes are calculated by using the magnetometer outputs Mx, My, and Mz. At the end, angle Hz of axis *Z* as to magnetic north is estimated.

The pitch, roll, and heading angles that were estimated in the previous step are used in Equation (1) (where *φ* = roll, *θ* = pitch, *ψ* = heading) in order to calculate the temporary rotation matrix ***Q***, which is the result of the product of 3 rotation matrices ***Q*_1_**, ***Q*_2_**, and ***Q*_3_**.

(1) $$Q\;=\;Q_{1}(\varphi )\cdot Q_{2}(\theta )\cdot Q_{3}(\psi )\;=\;\left[\begin{array}{ccc} \cos\theta \cdot \cos\psi & \sin\varphi \cdot \sin\theta \cdot \cos\psi -\cos\varphi \cdot \sin\psi & \cos\varphi \cdot \sin\theta \cdot \cos\psi +\sin\varphi \cdot \sin\psi \\ \cos\theta \cdot \sin\psi & \sin\varphi \cdot \sin\theta \cdot \sin\psi +\cos\varphi \cdot \cos\psi & \cos\varphi \cdot \sin\theta \cdot \sin\psi -\sin\varphi \cdot \cos\psi \\ -\sin\theta & \sin\varphi \cdot \cos\theta & \cos\varphi \cdot \cos\theta \end{array}\right]\;$$

The temporary rotation matrix ***Q*** is composed of elements describing the unit vector coordinates of the sensor system axes. The 3 columns and 3 rows of the rotation matrix represent the coordinates of the sensor unit vector as to the ground coordinate system and the sensor coordinate system, respectively. Specifically, the third row represents the attitude of the gravity acceleration unit vector having coordinates (*R*_31_, *R*_32_, *R*_33_) as to the IMU system. By taking into account the above physical interpretation, the final rotation matrix ***DCM*** (Equation (2)) is formed by rearranging the columns of the temporary matrix ***Q***.

(2) $$DCM\;=\;\left[\begin{array}{ccc} R_{11} & R_{12} & R_{13}\\ R_{21} & R_{22} & R_{23}\\ R_{31} & R_{32} & R_{33} \end{array}\right]\;$$

For this specific example, the first column of the temporary matrix is column c_3_ of the ***DCM***, the second is c_1_, and the third is c_2_ (Figure 1).

**Figure 2:**
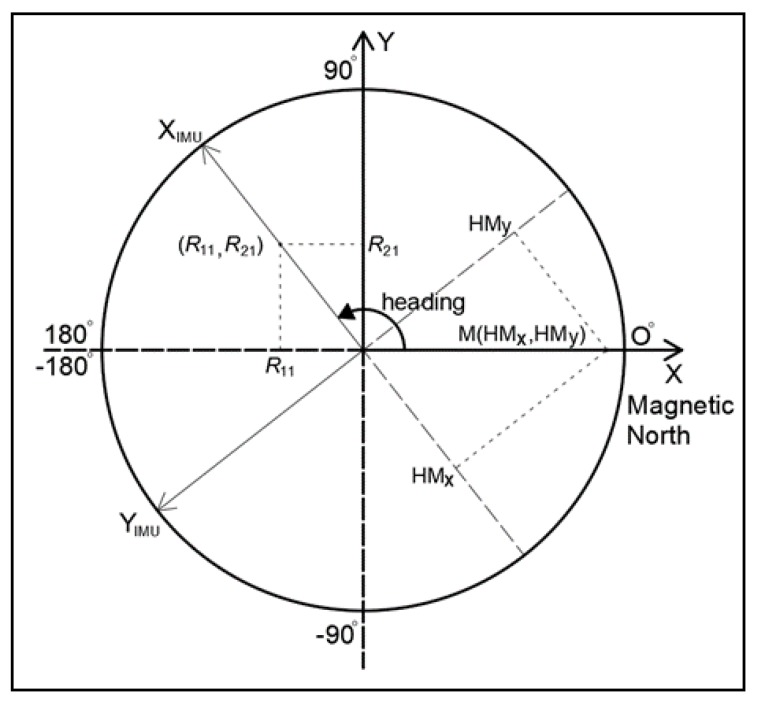
Connection of the sensor coordinate system with the ground reference system in the horizontal plane.

The connection of the sensor body frame and the ground reference coordinate system in the horizontal plane is accomplished by using the horizontal components of the magnetic field intensity (e.g., HMx, HMy) and the first 2 column elements of the rotation matrix (e.g., *R*_11_ and *R*_21_). The 2 coordinate systems are connected by the estimated heading angle, as presented in Figure 2.

### 2.2. Dynamic Estimation of the 3D Orientation

In low-dynamic movements, gyroscopes are used to smooth the results of the navigation parameters and to reduce the noise of both accelerometer and magnetometer outputs. In high-dynamic movements, low-cost gyroscopes can be used alone for a short time in order to maintain the representation of the 3D orientation of the sensor axis system. However, after a few seconds, additive errors caused by gyroscope drift create large errors and inhibit their further use.

Continuous knowledge of the attitude and heading of the inertial measurement unit’s axis system requires constant updating of the rotation matrix with respect to time. This can be realized with the aid of the matrix relationship shown in Equation (3) [24,30,31]. This relationship connects 2 successive 3D orientation positions of the sensor coordinate system ***DCM*** (*t*) and ***DCM*** (*t* + *Δt*) as a function of the angles *ω*_x_, *ω*_y_, *ω*_z_ traveled around the 3 respective axes.

(3) $$DCM(t+\Delta t)=DCM(t)\left[\begin{array}{ccc} 1 & -\omega_{z} & \omega_{y}\\ \omega_{z} & 1 & -\omega_{x}\\ -\omega_{y} & \omega_{x} & 1 \end{array}\right]\;$$

**Figure 3:**
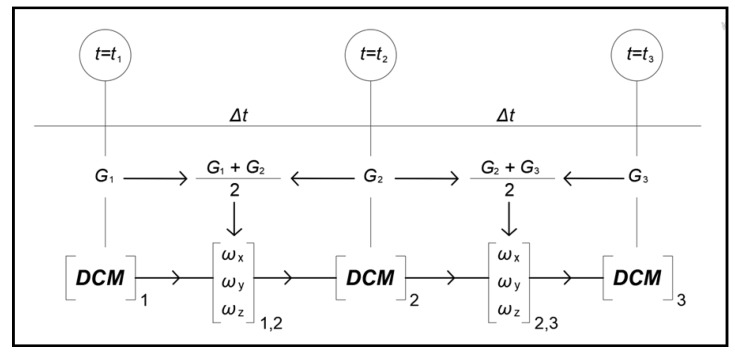
Angle calculation and update of rotation matrix by using output of gyroscopes.

The angles *ω*_x_, *ω*_y_, *ω*_z_ traveled in space as to the inertial measurement unit coordinate system result from the completion of the average values of the gyroscope sensor output ***G*** (*G*_x_, *G*_y_, *G*_z_) with respect to integration time *Δt*. Then, the rotation matrix ***DCM*** is updated, as presented in the flowchart of Figure 3.

**Figure 4:**
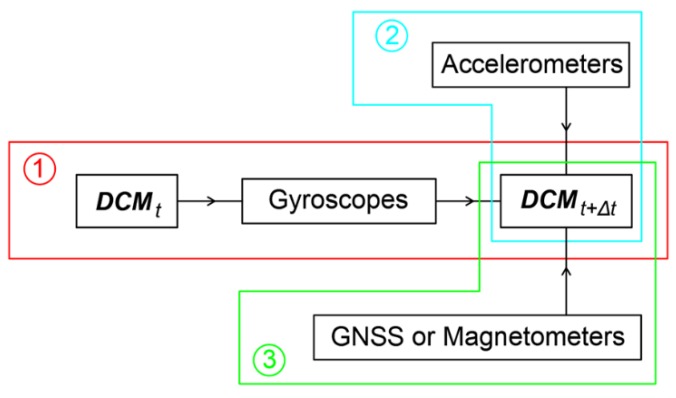
General flowchart for calculating the rotation matrix by combining outputs from all available sensors.

In real time and in non-static conditions, the rotation matrix that represents the 3D orientation of the sensor axis system is calculated by involving the outputs acquired by gyroscope, accelerometer, and magnetometer sensors. The general flowchart for calculating the rotation matrix, according to the type of sensor that the output is acquired from, is presented in Figure 4.

In procedure 1 (Figure 4), the ***DCM*** between 2 time points is calculated by using gyroscopes that usually have the largest measuring frequency. In procedure 2, whenever outputs from the accelerometer sensors are available, they are applied to the ***DCM*** to correct the tilts as to the horizontal level. Finally, in procedure 3, the magnetometer outputs that usually have the lowest operation frequency are used to correct the horizontal angle as to magnetic north.

Before using the raw sensor outputs to restore the ***DCM***, corrections in the form of factors and additive constants that emerge by calibration procedures have to be applied to the raw data outputs [14].

### 2.3. Mathematical Formulation of the Fusion Method Using Kalman Filter

The optimal design objectives of a Kalman filter method should take into account all error sources and must contain an accurate description of the system’s dynamics. The computational power when using the Kalman filter, according to [32], is associated with matrix inversion and is proportional to n^3^ (where n is the matrix dimension). The Kalman filter equation system, with more than 3 unknown parameters, may exhaust the available computing power of a portable device. For this reason, techniques that can reduce the computational load must be applied whenever possible. Additionally, the best strategy in designing a mathematical model that correlates measurements and the vector of unknowns is to formulate appropriate linear, rather than nonlinear, equations in the simplest possible form. When nonlinear equations are used, linearization and expansion of the Kalman filter are required (extended Kalman filter [33]). In this case, more complex equations are formed and additional calculations are needed. Furthermore, reduced precision should be expected, as only the first term of a Taylor series expansion is used.

In this research, the framework for designing the methodology for the synthesis of gyroscope, accelerometer, and magnetometer outputs using the Kalman filter was defined by the need to create an algorithm that gives precise results without high computational cost. For these reasons, a linear mathematical model was created and parameters, such as the gyroscope and accelerometer biases and scale factors, were treated as constants and were precalibrated in an external procedure [14]. In this way, it became possible to limit the unknown parameters (states) to 3.

There are many formulations of the Kalman filter, and the one that was adopted in this work is described in detail in [34]. The operation of the Kalman filter requires the design of 2 mathematical models; these are the process model, or state equation (see Equation (4)), and the measurement model, or observation equation (see Equation (5)). The state equation is of the form

(4) $$x_{k}=A\cdot x_{k-1}+B\cdot u_{k-1,\;k}+w_{k-1,\;k}\;$$

where ***x*** is the state matrix (vector of unknowns), ***A*** is a transition matrix, ***B*** is the control matrix, ***u*** is a known exogenous control input (in our case the output of the gyroscopes), ***w*** is a vector of the entire process noise, and *k*, *k* – 1 are the time consecutive points (epochs).

The observation equation reads as

(5) $$y_{k}=H_{k}\cdot x_{k}+z_{k}\;$$

where ***y*** is the vector of observations (in our case the accelerometer output), ***Η*** is the observation matrix, ***x*** is the vector of unknowns, ***z*** is the vector of measurement noise, and *k* is the time point (epoch).

Since the equations of the Kalman filter are fully documented in [34], it is sufficient to formulate the state and observation equations so that the mathematical model of the considered fusion method is fully defined. The complete formulation of the Kalman filter mathematical model of this study is presented by Equations (6) and (7).

The proposed fusion method is divided into 2 parts: (a) the combination of accelerometer/gyroscope outputs, and (b) the combination of magnetometer/gyroscope outputs.

In the first part, the method combines outputs from accelerometers and gyroscopes, thereby ensuring updated attitude information of the axis system. 

The state equation (see Equation (6)) is derived from Equations (3) and (4) and connects the elements of the third row of the rotation matrix (*R*_31_, *R*_32_, *R*_33_) between 2 consecutive epochs (*k* − 1, *k*), through measurements of the gyroscopes (*G*_x_, *G*_y_, *G*_z_) and considering integration time *Δt*.

(6) $${\left[\begin{array}{c} R_{31}\\ R_{32}\\ R_{33} \end{array}\right]}_{k}=\left[\begin{array}{ccc} 1 & 0 & 0\\ 0 & 1 & 0\\ 0 & 0 & 1 \end{array}\right]\cdot {\left[\begin{array}{c} R_{31}\\ R_{32}\\ R_{33} \end{array}\right]}_{k-1}+{\left[\begin{array}{ccc} 0 & -R_{33}\cdot \Delta t & R_{32}\cdot \Delta t\\ R_{33}\cdot \Delta t & 0 & -R_{31}\cdot \Delta t\\ -R_{32}\cdot \Delta t & R_{31}\cdot \Delta t & 0 \end{array}\right]}_{k-1}\cdot {\left[\begin{array}{c} G_{x}\\ G_{y}\\ G_{z} \end{array}\right]}_{k-1,\;k}+{\left[\begin{array}{c} w_{x}\\ w_{y}\\ w_{z} \end{array}\right]}_{k-1,\;k}\;$$

The observation equation (Equation (7)) constitutes the output of the accelerometers (*A*_x_, *A*_y_, *A*_z_), the third row of the rotation matrix (*R*_31_, *R*_32_, *R*_33_), and the vector (*z*_x_, *z*_y_, *z*_z_) of measurement noise.

(7) $${\left[\begin{array}{c} A_{x}\\ A_{y}\\ A_{z} \end{array}\right]}_{k}=\left[\begin{array}{ccc} 1 & 0 & 0\\ 0 & 1 & 0\\ 0 & 0 & 1 \end{array}\right]\cdot {\left[\begin{array}{c} R_{31}\\ R_{32}\\ R_{33} \end{array}\right]}_{k}+{\left[\begin{array}{c} z_{x}\\ z_{y}\\ z_{z} \end{array}\right]}_{k}\;$$

The constituents (*w*_x_, *w*_y_, *w*_z_) that represent procedure noise must be estimated at each epoch *k*. The equations for calculating the normalized components of acceleration are given in Equation (8).

(8) $$\begin{array}{l} R_{31}{}_{{}_{k}}=R_{31_{{}_{k-1}}}-R_{33_{{}_{k-1}}}\cdot \Delta t\cdot G_{y_{{}_{k-1,\;k}}}+R_{32_{{}_{k-1}}}\cdot \Delta t\cdot G_{z_{k-1,\;k}}\\ R_{32_{{}_{k}}}=R_{32_{{}_{k-1}}}+R_{33_{{}_{k-1}}}\cdot \Delta t\cdot G_{x}{}_{{}_{{}_{k-1,\;k}}}-R_{31_{{}_{k-1}}}\cdot \Delta t\cdot G_{z}{}_{{}_{{}_{k-1,\;k}}}\\ R_{33_{{}_{k}}}=R_{33_{{}_{k-1}}}-R_{32_{{}_{k-1}}}\cdot \Delta t\cdot G_{x}{}_{{}_{{}_{k-1,\;k}}}+R_{31_{{}_{k-1}}}\cdot \Delta t\cdot G_{y}{}_{{}_{{}_{k-1,\;k}}} \end{array}\;$$

The elements of the noise matrix ***w*** within the process model of Equation (6) are calculated by applying the propagation covariance law [35] to Equation (8). This is carried out using the observations of the outputs obtained by the gyroscope sensors and their sampling variances σ^2^ (.) as follows:

(9) $$w_{i}{}^{2}=\sigma^{2}(R_{3i})={\left(\frac{\partial R_{3i}}{\partial G_{x}}\right)}_{}^{2}\cdot \sigma^{2}(G_{x})+{\left(\frac{\partial R_{3i}}{\partial G_{y}}\right)}_{}^{2}\cdot \sigma^{2}(G_{y})+{\left(\frac{\partial R_{3i}}{\partial G_{z}}\right)}_{}^{2}\cdot \sigma^{2}(G_{z})\;$$

The noise ***z****_k_* of the measurement model (see Equation (9)) can be estimated as a function of the accelerometer sensor operation [23] or simply by using the variability of the output of the accelerometers per axis acquired from large samples in a static condition.

In the second part, the method performs a correction of the horizontal orientation. Depending on the axis that has the largest value, the first 2 column elements of the rotation matrix are used. For example, in case the *Z*-axis is upward, then the *X*-axis is used to determine the heading of the system. Therefore, the process model and the observation equation of the Kalman filter are represented by Equations (10) and (11).

(10) $${\left[\begin{array}{c} R_{11}\\ R_{21} \end{array}\right]}_{k}=\left[\begin{array}{cc} 1 & 0\\ 0 & 1 \end{array}\right]\cdot {\left[\begin{array}{c} R_{11}\\ R_{21} \end{array}\right]}_{k-1}+{\left[\begin{array}{cc} -R_{13}\cdot \Delta t & R_{12}\cdot \Delta t\\ -R_{23}\cdot \Delta t & R_{22}\cdot \Delta t \end{array}\right]}_{k-1}\cdot {\left[\begin{array}{c} G_{y}\\ G_{z} \end{array}\right]}_{k-1,\;k}+{\left[\begin{array}{c} w_{y}\\ w_{z} \end{array}\right]}_{k-1,\;k}\;$$

(11) $${\left[\begin{array}{c} R_{11}\\ R_{21} \end{array}\right]}_{k}^{magnetometers}=\left[\begin{array}{cc} 1 & 0\\ 0 & 1 \end{array}\right]\cdot {\left[\begin{array}{c} R_{11}\\ R_{21} \end{array}\right]}_{k}^{IMU}+{\left[\begin{array}{c} z_{x_{p}}\\ z_{y_{p}} \end{array}\right]}_{k}\;$$

In Equations (10) and (11), the elements of the noise matrix ***w*** are estimated in the same way as described before in the first part of the method. In addition, the elements $z_{x_{p}}$, $z_{y_{p}}$ of the noise matrix ***z_k_*** can be estimated by using the propagation covariance law, applied to the equations for calculating the projections of the magnetometer measurements on the plane [27]. 

Alternatively, if the *X*-axis is upward and the *Y*-axis is used to determine the system’s orientation, the process model and the measurement model are formed in Equations (12) and (13).

(12) $${\left[\begin{array}{c} R_{12}\\ R_{22} \end{array}\right]}_{k}=\left[\begin{array}{cc} 1 & 0\\ 0 & 1 \end{array}\right]\cdot {\left[\begin{array}{c} R_{12}\\ R_{22} \end{array}\right]}_{k-1}+{\left[\begin{array}{cc} R_{13}\cdot \Delta t & -R_{11}\cdot \Delta t\\ R_{23}\cdot \Delta t & -R_{21}\cdot \Delta t \end{array}\right]}_{k-1}\cdot {\left[\begin{array}{c} G_{x}\\ G_{z} \end{array}\right]}_{k-1,\;k}+{\left[\begin{array}{c} w_{x}\\ w_{z} \end{array}\right]}_{k-1,\;k}\;$$

(13) $${\left[\begin{array}{c} R_{12}\\ R_{22} \end{array}\right]}_{k}^{magnetometers}=\left[\begin{array}{cc} 1 & 0\\ 0 & 1 \end{array}\right]\cdot {\left[\begin{array}{c} R_{12}\\ R_{22} \end{array}\right]}_{k}^{IMU}+{\left[\begin{array}{c} z_{x_{p}}\\ z_{y_{p}} \end{array}\right]}_{k}\;$$

At the end, when the *Y*-axis is upward and the *Z*-axis is used, the equations are given as:

(14) $${\left[\begin{array}{c} R_{13}\\ R_{23} \end{array}\right]}_{k}=\left[\begin{array}{cc} 1 & 0\\ 0 & 1 \end{array}\right]\cdot {\left[\begin{array}{c} R_{13}\\ R_{23} \end{array}\right]}_{k-1}+{\left[\begin{array}{cc} -R_{12}\cdot \Delta t & R_{11}\cdot \Delta t\\ -R_{22}\cdot \Delta t & R_{21}\cdot \Delta t \end{array}\right]}_{k-1}\cdot {\left[\begin{array}{c} G_{x}\\ G_{y} \end{array}\right]}_{k-1,\;k}+{\left[\begin{array}{c} w_{x}\\ w_{y} \end{array}\right]}_{k-1,\;k}\;$$

(15) $${\left[\begin{array}{c} R_{13}\\ R_{23} \end{array}\right]}_{k}^{magnetometers}=\left[\begin{array}{cc} 1 & 0\\ 0 & 1 \end{array}\right]\cdot {\left[\begin{array}{c} R_{13}\\ R_{23} \end{array}\right]}_{k}^{IMU}+{\left[\begin{array}{c} z_{x_{p}}\\ z_{y_{p}} \end{array}\right]}_{k}\;$$

The ***DCM*** that results from the process model, due to cumulative gyroscope errors, presents the problems of the nonrectangularity and nonnormality of its rows-vectors.

In the nonrectangularity problem, the row elements that represent the unit coordinates as to the sensor reference coordinate system are not perpendicular to each other. If ***X***, ***Y***, and ***Z*** are the rows-vectors of the ***DCM***, then their interior product will not be zero. That means that the vectors are not perpendicular to each other, but there is a value that results from Equation (16).

(16) error= X⋅Y 

This error has to be shared by both vectors (Equation (17)) so at the end they will be vertical to each other [24].

(17) $$X_{\mathrm{ortho}}=X-\frac{error}{2}Y,\;Y_{\mathrm{ortho}}=Y-\frac{error}{2}Χ\;$$

The third vector ***Z*** results from using the exterior product of vectors ***X*** and ***Y*** (Equation (18)), which represents a vector vertical to both vectors.

(18) $$Z_{\mathrm{ortho}}=\;X_{\mathrm{ortho}}\times Y_{\mathrm{ortho}}\;$$

The nonnormality problem concerns the fact that the magnitude of each row of the ***DCM*** is not equal to the unit as it should be, because they have to be unit vectors. Normalization of the ***DCM*** is accomplished by dividing each row component by the magnitude of the corresponding vector. For example, for the 3 elements of the first row–vector (***u***_1_) of the ***DCM***, the normalization will be accomplished by (*R*_11_/|***u***_1_|*R*_12_/|***u***_1_|*R*_13_/|***u***_1_|), where |***u***_1_| is the magnitude of vector ***u***_1_. On the other hand, the normalization and rectangularity of the unit axes of the ***DCM*** that are estimated by the measurement model are ensured using Equation (1).

## 3. Evaluation of the Method and Discussion

The method was practically evaluated in two discrete steps. In the first step, a classic low-cost MEMS strap-down system sensor was used with the help of a custom software utility developed in order to implement, test, and evaluate the fusion method discussed in this paper. In the second step, a mobile tablet device containing all the appropriate MEMS sensors and, additionally, a camera sensor was used to test and evaluate the method in an AR application developed from scratch.

### 3.1. Development and Evaluation of the Fusion Method Using a Low-Cost Sensor System and a Custom Software Utility

**Figure 5:**
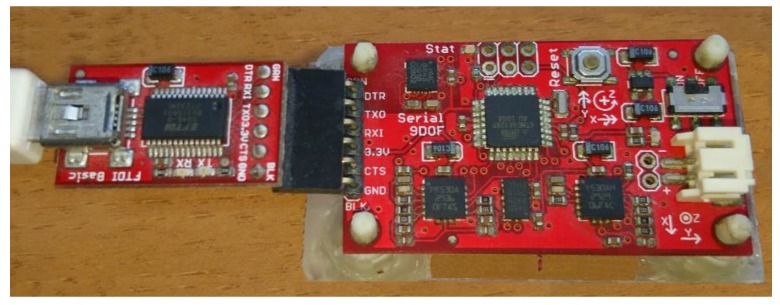
Low-cost microelectromechanical systems (MEMS) strap-down sensor system, SparkFun Razor 9DOF, with a serial-to-USB adapter mounted on an improvised basis.

The low-cost MEMS strap-down sensor system that was used to implement and evaluate the fusion method was the SparkFun Razor 9DOF. The specific sensor system includes three gyroscopes, three accelerometers, and three magnetometer sensors in a three-rectangle layout (Figure 5). The inertial sensors (accelerometers and gyroscopes) of the specific low-cost inertial measurement unit work at a nominal frequency of 100 Hz and the magnetometer sensors operate at 20 Hz. Transmission of the measurements by the sensors to a connected host computer was carried out via a serial connection and a USB adapter (Figure 5).

**Figure 6:**
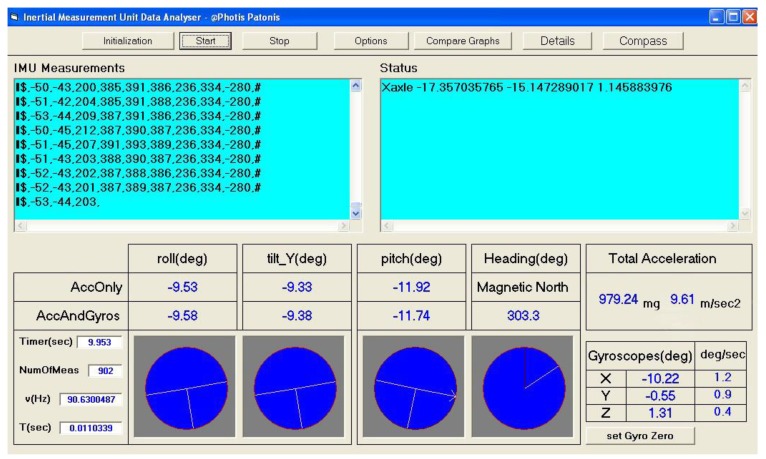
Inertial measurement unit data analyzer software utility.

A software utility called Inertial Measurement Unit Data Analyzer was developed in the Visual Basic programming language in order to debug the implementation and evaluate the fusion method. The utility captures the outputs acquired from the IMU and enhances them by applying corrections that emerge from a previous calibration procedure [14]. Next, the 3D orientation of the IMU body frame is estimated in real time and is expressed in the form of navigation angles, rotation matrix, and quaternions, while the navigation parameters are represented in numerical and visual form, as shown in Figure 6. The user can visually observe the results of the fusion method in static and kinematic conditions. In this way, the equations of the mathematical model are tested and verified by monitoring the performance of different settings applied programmatically. 

In the static condition, the evaluation refers to the observation of the relative variation of the pitch, roll, and heading angles and whether they remain constant around a fixed (true) value over a long time. Thus, the efficiency of the dynamic weights assigned to the output of the sensors by the Kalman filter are checked. In the case where the accelerometer/magnetometer outputs will be incorrectly overweighted, correct absolute (true) values will be observed but with large relative variations. Otherwise, when incorrectly higher weights are given to the gyroscope output, the pitch, roll, and heading angles will present small variations. In addition, the absolute (true) angles will gradually change over time with a rate of change proportional to the drift effect that characterizes the output by low-cost gyroscopes.

**Figure 7:**
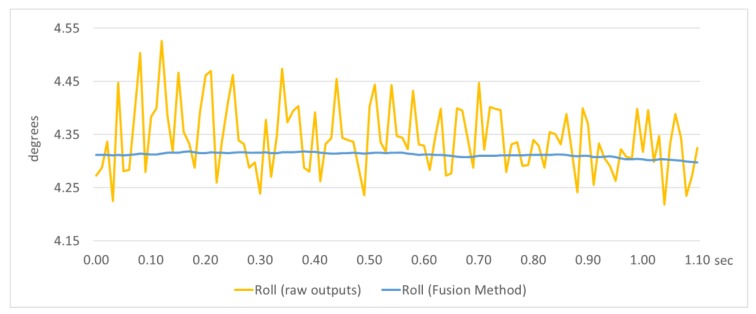
Roll angle estimation by raw outputs and the fusion method in static condition.

After the practical evaluation in a static condition, it was concluded that the variations of pitch, roll, and heading angles were almost negligible and they remained constant at their true values. In the optical representation of the navigation angles (Figure 6), the movement of the graphics, when the sensor is steady, is imperceptible to the naked eye. Specifically, the variation in the navigation angles before applying the fusion method was around 3° for the heading angle and 0.2° for the pitch/roll angles, and after applying the method it was limited to 0.2° and 0.007°, respectively. An example of the variation of roll angle is presented in Figure 7, where in a sample output in static condition, the roll angle is estimated using raw outputs and outputs provided by the fusion method. 

Moreover, the order of magnitude of the angle variation is less than the accuracy of commercial low-cost IMU devices [36]. Notably, the accuracy of commercial low-cost IMU devices is 1° for the heading and 0.2° for the pitch/roll angles. In this way, the variation of navigation angles does not degrade the accuracy of these sensors, allowing the system to achieve optimal performance.

**Figure 8:**
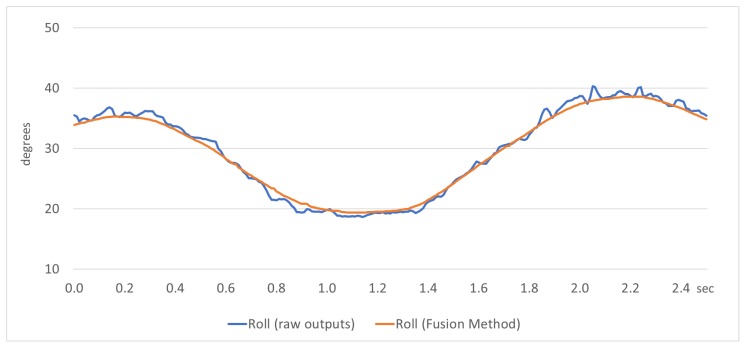
Roll angle estimation by raw outputs and the fusion method in kinematic condition.

In kinematic conditions concerned mainly with rotational movements of the sensor body frame, the visual results of the actual movement of the device (see Figure 6) showed that the fusion method provides directly smooth results for the navigation parameters. In Figure 8, a comparison sample is presented, where the roll angle is estimated using raw outputs and outputs provided by the fusion method. It is clear that the fusion method smooths the raw outputs by the accelerometer sensors, providing satisfactory results for the navigation parameter examined.

**Figure 9:**
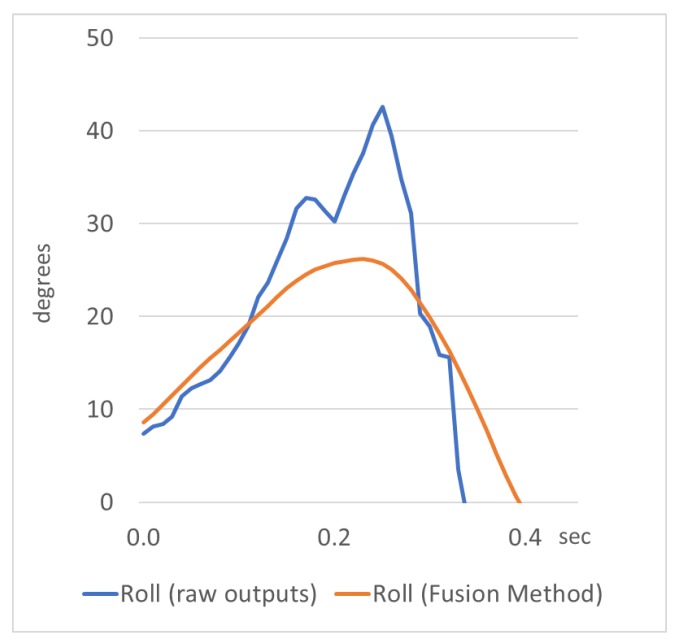
Absorption of sudden movements of the mobile device using the fusion method.

Furthermore, practical evaluation of the fusion method showed that major disorders of the accelerometer measurements caused by irregular or sudden movements of the sensor body frame were sufficiently absorbed, as shown in Figure 9.

### 3.2. Evaluation of the Fusion Method on a Mobile Device and an AR Application 

The fusion method was implemented in the Java programming language on a mobile device powered by Android, and was tested in an AR application developed from scratch [7]. The Eclipse LUNA integrated development environment was used to debug the code and implement the AR functionality. The mobile device used was a 12 inch tablet equipped with all necessary MEMS sensors for attitude and heading estimation and the computational power to meet the requirements of an AR application. The technical specifications of the mobile device and its sensors are shown in Table 1.

**Figure 10:**
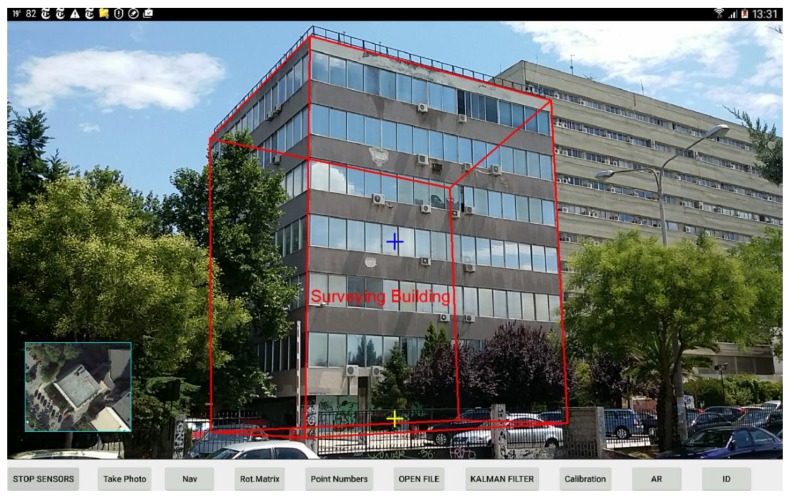
AR application on the mobile device display.

The AR application displays on the mobile’s screen georeferenced digital spatial data with descriptive labels along with the camera’s preview, shown in red in Figure 10. Furthermore, utilities including a static targeting cross (blue cross) and an inertial cross (yellow cross) indicating horizontal level and tilts of the camera, respectively, were implemented. In the bottom-left corner of the AR application, a frame with a thumbnail of the top view of the object targeted is also displayed.

The fusion method operates in a Service [37] in the Android background environment independent from the main application. Communication between the main application and the Service is accomplished via Android Interface Definition Language (AIDL) [38]. The frequency of data transmission from the Service to the main application was programmatically set to 10 Hz, the same as the refresh rate of the graphics drawn to the mobile device’s display.

The operation of the AR application was tested gradually in all available sensors operating frequency modes, as presented in Table 2.

Table 2 shows the sensor frequency rates at all operating modes and the total events per second (sensor outputs) that cause an equal number of iterations as the Kalman filter approach. Finally, the CPU usage of the mobile device processor is given for every case. It is clear that the application does not require significant computational power, even if the highest operating frequencies are used. In the worst-case scenario, 15% of the total power of the processor is used. It should be noted that this percentage refers to the execution of the AR application and not to the fusion method itself. This means that the fusion method’s central processing unit (CPU) usage will be even lower.

In the general case, AR applications operate satisfactorily on the Game rate mode using 50 Hz for all sensors of the specific mobile device. However, it was considered necessary to test the fusion method at higher possible operating rate frequencies to examine the possibility of using the method in kinematic applications. Therefore, the attitude and heading representation parameters were calculated using gyroscopes at 200 Hz, accelerometers at 100 Hz, and magnetometers at 50 Hz. The impression from the fusion method performance on the AR application for the specific mobile device, the given AR data load, and when the sensors operate at the highest possible frequency was that it worked efficiently. Indeed, the application worked without causing any noticeable delay in the overall performance of the mobile device, practically verifying the CPU usage values given in Table 1. Considering the high operating frequencies and the modest technical features of this mobile device, it is certain that the method will work efficiently on any modern mobile smartphone.

As far as the practical evaluation of the fusion method in the AR application is concerned, the findings from the low-cost MEMS strap-down sensor system and the software utility were fully verified. The fusion method was tested in random regular and irregular movements of the sensor body frame and worked efficiently in every case. Specifically, the AR graphics moved smoothly and responded immediately to the sensor movements. A specialized test focusing on sudden movements was carried out and again revealed that the fusion method effectively absorbed their effects.

The absolute accuracy of the AR elements location as to the real-world objects is difficult to test due to the low performance in positioning caused by the low-cost Global Navigation Satellite System (GNSS) receivers with which mobile devices are equipped. However, the application offers a pose estimation correction tool that can adjust the positions of AR elements to real-world objects by arithmetically or graphically inputting the position and heading of the camera as to the ground coordinate system. The result of the adjustment is extremely precise (Figure 10), and in this way the repeatable performance of the fusion method can be evaluated. Specifically, this was tested by repeatedly targeting the same object and checking the AR element position as to the real-world object. We found that repeatability was satisfactory for general use of an AR application.

Finally, the AR application was tested in all possible 3D orientation positions to verify that the methodology developed deals efficiently with the “gimbal lock” problem. The rotation matrix (Equation (3)) was estimated by the system for a rotation of 360° per axis divided into eight sections for each case. The elements of the rotation matrices are shown in Table 3.

**Figure 11:**
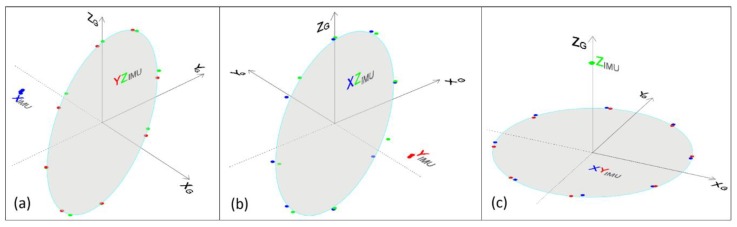
Unit vector coordinates of the IMU’s axes as to the ground reference system.

The 3D representation of the unit coordinates presented in Table 3 as to the ground coordinate system is displayed in Figure 11. In case a, where the rotation is about the *X*-axis of the IMU (X_IMU_), the coordinates of the X_IMU_ axis are concentrated around a limited area, while the coordinates of the Y_IMU_ and Z_IMU_ axes are distributed evenly and uniformly around a flat surface (YZ_IMU_). The same conclusion can be drawn for the two other cases, b and c, of Figure 11. In this way, it is proved that the methodology works flawlessly in all cases bypassing the above-mentioned singularity problem.

## 4. Conclusions and Future Work

The proposed fusion method successfully combines outputs acquired by low-cost accelerometers, gyroscopes, and magnetometers to estimate the optimal 3D orientation of the sensor axes. The method provides Euler–Cardan angles and rotation matrix for attitude and heading representation in real time.

The fusion method provides results that present minimum variance when the sensors operate in a static condition and a smooth change of the 3D orientation parameters in kinematic conditions, while the effects of sudden movements of the sensor body frame are effectively absorbed. Furthermore, the method works flawlessly in any 3D orientation of the system, dealing efficiently with the “gimbal lock” problem.

Concerning the fusion method performance on mobile devices, it was found that for the specific AR data load and the available hardware, processing of the sensor output, operating at the highest possible frequency, is accomplished efficiently without overusing the available computational power. The AR graphics respond directly and smoothly to random regular and irregular movements, while the repeatability of drawing AR graphics in relation to real-world objects works satisfactorily. It can also be noticed that the fusion method can be used efficiently on AR and general representation software applications that use low-cost MEMS sensors on mobile devices.

The pose estimation of a mobile device is the core of geomatics and location-based AR applications. The method developed in this paper can be used alternatively for sensor fusion in estimating the 3D orientation of the mobile device, connecting spatial digital data with real-world objects. Future work will be an extension of the AR application presented in this paper, including more sophisticated features applicable to geomatics applications in the field of photogrammetry. In these cases, the limited accuracy in pose estimation due to the low-cost sensors is not an obstacle, especially when speed of surveying is more important than accuracy. Alternatively, the pose estimation calculated directly by the sensor measurements can be used as initial approximate values in hybrid robust pose estimation through visual/GNSS mixing.

## Figures and Tables

**Table 1:** Technical specifications of the mobile device used in the augmented reality (AR) application. CPU, central processing unit; RAM, random access memory; CMOS, complementary metal-oxide semiconductor.

**Model**	12” tablet
**Storage**	32 GB
**CPU**	Quad 2.3 GHz
**Primary Camera**	CMOS, 8 MP (3264 × 2448 pixels)
**RAM**	3 GB
**Display**	TFT 2560 × 1600 (WQXGA) 12.2”, 16 million colors
**Accelerometers**	Type: BMI055, Mfr: Bosch Sencortec, Ver: 1, Resolution: 0.038, Rate: 100 Hz, Unit: m/s^2^
**Gyroscopes**	Type: BMI055, Mfr: Bosch Sencortec, Ver: 1, Resolution: 2.66316E-4, Rate: 200 Hz, Unit: rad/s
**Magnetometers**	Type: AK8963C magnetic field sensor, Mfr: Asahi, Kasei Microdevices, Ver: 1, Resolution: 0.060, Rate: 50 Hz, Unit: μT

**Table 2:** Application CPU usage per sensors operating rate mode.

Operating Rate Mode	Sensor	Rate (Hz)	Type of Use	Events/s	CPU Usage (%)
NORMAL	Accelerometer Gyroscope Magnetometer	15 5 5	Rate (default) suitable for screen orientation changes	25	9
UI	Accelerometer Gyroscope Magnetometer	15 15 15	Rate suitable for user interface	45	10
GAME	Accelerometer Gyroscope Magnetometer	50 50 50	Rate suitable for games	150	13
FASTEST	Accelerometer Gyroscope Magnetometer	100 200 50	Get sensor data as fast as possible	350	15

**Table 3:** Elements of the rotation matrix (unity values) of the inertial measurement unit (IMU) axes when rotated 360° around its axes.

		*R*_11_	*R*_21_	*R*_31_	*R*_12_	*R*_22_	*R*_23_	*R*_31_	*R*_32_	*R*_33_
Rotation around *X*_IMU_ axis	1	−0.9677	−0.2519	0.0075	0.2520	−0.9677	0.0016	0.0069	0.0034	1.0000
	2	−0.9666	−0.2564	0.0047	0.2043	−0.7588	0.6184	−0.1550	0.5987	0.7858
	3	−0.9712	−0.2379	0.0100	0.0324	−0.0907	0.9953	−0.2359	0.9670	0.0958
	4	−0.9648	−0.2629	0.0087	−0.1361	0.5273	0.8387	−0.2251	0.8080	−0.5446
	5	−0.9658	−0.2592	0.0089	−0.2592	0.9658	−0.0031	−0.0078	−0.0053	−1.0000
	6	−0.9621	−0.2724	−0.0124	−0.2100	0.7690	−0.6038	0.1740	−0.5783	−0.7971
	7	−0.9678	−0.2516	0.0068	−0.0076	0.0021	−1.0000	0.2516	−0.9678	−0.0040
	8	−0.9609	−0.2767	0.0093	0.1981	−0.7106	−0.6751	0.1934	−0.6469	0.7376
Rotation around *Y*_IMU_ axis	1	−0.9781	−0.2078	0.0101	0.2079	−0.9782	−0.0007	0.0100	0.0014	0.9999
	2	−0.7457	−0.1457	0.6501	0.1863	−0.9825	−0.0065	0.6397	0.1163	0.7598
	3	−0.0274	−0.0149	0.9995	0.2218	−0.9751	−0.0085	0.9747	0.2214	0.0300
	4	0.5493	0.0849	0.8313	0.1682	−0.9857	−0.0105	0.8185	0.1456	−0.5557
	5	0.9734	0.2285	0.0137	0.2287	−0.9735	−0.0088	0.0113	0.0117	−0.9999
	6	0.6635	0.1952	−0.7223	0.2716	−0.9624	−0.0107	−0.6972	−0.1891	−0.6915
	7	−0.0141	0.0002	−0.9999	0.2889	−0.9574	−0.0042	−0.9573	−0.2889	0.0134
	8	−0.7845	−0.1990	−0.5873	0.2443	−0.9697	0.0022	−0.5700	−0.1418	0.8094
Rotation around *Z*_IMU_ axis	1	−0.9722	−0.2340	0.0077	0.2340	−0.9722	−0.0020	0.0080	−0.0001	1.0000
	2	−0.8729	0.4878	0.0108	−0.4878	−0.8730	0.0022	0.0105	−0.0034	0.9999
	3	−0.3076	0.9515	0.0100	−0.9515	−0.3076	−0.0037	−0.0005	−0.0107	0.9999
	4	0.4668	0.8841	0.0221	−0.8842	0.4671	−0.0087	−0.0180	−0.0154	0.9997
	5	0.9621	0.2717	0.0233	−0.2718	0.9624	0.0025	−0.0218	−0.0088	0.9997
	6	0.8910	−0.4536	0.0194	0.4537	0.8912	0.0014	−0.0179	0.0075	0.9998
	7	0.3463	−0.9380	0.0186	0.9380	0.3464	0.0072	−0.0132	0.0150	0.9998
	8	−0.4209	−0.9071	0.0099	0.9071	−0.4208	0.0092	−0.0042	0.0128	0.9999
